# Semiflexible Polymers Interacting with Planar Surfaces: Weak versus Strong Adsorption

**DOI:** 10.3390/polym12020255

**Published:** 2020-01-22

**Authors:** Andrey Milchev, Kurt Binder

**Affiliations:** 1 Institute of Physical Chemistry, Bulgarian Academy of Sciences, 1113 Sofia, Bulgaria; 2 Institut für Physik, Johannes Gutenberg-Universität Mainz, Staudinger Weg 9, D-55128 Mainz, Germany; kurt.binder@uni-mainz.de

**Keywords:** polymers, phase transitions, adsorption, chain rigidity, molecular dynamics

## Abstract

Semiflexible polymers bound to planar substrates by a short-range surface potential are studied by Molecular Dynamics simulations to clarify the extent to which these chain molecules can be considered as strictly two-dimensional. Applying a coarse-grained bead-spring model, the chain length *N* and stiffness κ as well as the strength of the adsorption potential $\epsilon_{wall}$ are varied over a wide range. The excluded-volume (EV) interactions inherent in this model can also be “switched off” to provide a discretized version of the Kratky–Porod wormlike chain model. We study both local order parameters (fraction *f* of monomers within the range of the potential, bond-orientational order parameter η) and the mean square gyration radius parallel, ${\langle R_{g}^{2}\rangle }_{\left|\right|}$, and perpendicular, ${\langle R_{g}^{2}\rangle }_{⊥}$, to the wall. While for strongly adsorbed chains EV has negligible effect on *f* and η, we find that ${\langle R_{g}^{2}\rangle }_{\left|\right|}$ is strongly affected when the chain contour length exceeds the persistence length. Monomer coordinates in perpendicular (⊥) direction are correlated over the scale of the deflection length which is estimated. It is found that f,η, and ${\langle R_{g}^{2}\rangle }_{⊥}$ converge to their asymptotic values with 1/N corrections. For both weakly and strongly adsorbed chains, the distribution functions of “loops”, “trains”, and “tails” are analyzed. Some consequences pertaining to the analysis of experiments on adsorbed semiflexible polymers are pointed out.

## 1. Introduction

Many macromolecules with linear chemical architecture are neither perfectly flexible nor entirely rigid-rod-like chain molecules, but exhibit instead only local stiffness and are called semiflexible. Within a coarse-grained description, such a polymer chain is modeled as a curve in continuous space $\vec{r}\left(s\right)$, with *s* a coordinate along the backbone of the macromolecule. The stiffness is due to a nonzero bending modulus κ, which is proportional to the persistence length $\ell_{p}$, describing the length along the contour over which the orientations of subsequent bonds (or tangent vectors along $\vec{r}\left(s\right)$, respectively) are correlated [1,2,3]. This Kratky–Porod model [3] (also-called Wormlike Chain (WLC) model) is widely accepted as a proper phenomenological description of semiflexible polymers, in particular, of biopolymers such as the double-stranded (ds) DNA [4], filamentous (F)-actin [5], etc.

When one deals with the liquid–crystalline order of semiflexible polymers in lyotropic solution [6,7], it is clear that a description in terms of two lengths only, the contour length *L* of the WLC and its persistence length, $\ell_{p}$, does not suffice: the effective chain diameter *D* controls the interchain repulsion and hence the possible onset of nematic order (see, e.g., [8,9,10,11]). However, for single semiflexible chains in d=3 dimensions (as they matter in dilute solutions), the excluded volume interactions due to nonzero *D* do not matter for large $\ell_{p}/D$, provided *L* is much smaller than $L^{*}=D{\left(\ell_{p}/D\right)}^{3}$ [12,13]. For this reason, the WLC model is broadly accepted as kind of a “gold standard” as far as the statistical mechanics of semiflexible chains is concerned.

Care is needed, however, when one deals with the adsorption of semiflexible polymers on planar surfaces [14,15,16,17,18,19,20]. When a polymer gets adsorbed, its conformation changes from three-dimensional to (quasi) two-dimensional [21,22,23]. Now, the WLC model implies that a polymer, which in d=3 exhibits a decay of the tangent–tangent correlation function with persistence length $\ell_{p}$, would exhibit in d=2 a corresponding decay with $2\ell_{p}$. In fact, adsorbed polymers on planar substrates are not at all strictly confined into a two-dimensional plane, one rather expects loops and tails. For flexible polymers, the structure of the adsorbed “pancake“ is rather a two-dimensional self-avoiding walk (SAW) of more or less spherical ”blobs“ of radius *r* attached to the surface whereby $r\propto \tau^{-\nu /\varphi }$. Here, τ≪1 is a variable denoting the relative distance from the adsorption transition point, using the temperature or the strength of the adsorption potential as control variable. The exponent ν≈0.588 is the Flory exponent [23] characterizing the size of a flexible polymer in bulk three-dimensional solution under good solvent conditions, and φ≈0.48 [24] is the so-called *crossover exponent* [22,25]. For Gaussian chains (e.g., appropriate for Θ-solvents [8,9]), the corresponding exponents are ν=1/2 [8,9], and φ=1/2 [21,22,23,24,25]. Thus, *r* exceeds the diameter *D* of the effective monomers for weak adsorption (τ≪1) while *r* is of the same order as *D* for the case of strong adsorption (τ>1). Only for the latter case can an orientation of the bond vectors between subsequent effective monomers predominantly parallel to the adsorbing substrate be anticipated.

For semiflexible polymers with $\ell_{p}\gg D$, the regime of weak adsorption is predicted to be much narrower [15], $\tau \leq \tau^{**}\propto {\left(\Delta /\ell_{p}\right)}^{2/3}$, where Δ is the range of the adsorption potential (we assume for simplicity an adsorption potential of short range Δ of the same order as *D*). Note that, for adsorbed semiflexible polymers, long loops for $\tau >\tau^{**}$ are expected to be very rare, but if they occur they must bend away from the substrate over distances larger than $\ell_{p}$ [15]. However, short wormlike loops, which are nearly parallel to the surface, are still expected in the regime of strong adsorption, their length being gradually suppressed with increasing τ. The fraction of monomers *f* within the potential range Δ is, however, predicted [15] to be close to unity already when τ exceeds $\tau^{*}\propto {\left(\Delta /\ell_{p}\right)}^{4/3}\ll \tau^{**}$.

These predictions imply that an adsorbed semiflexible polymer is not identical to a strictly two-dimensional chain confined in a plane parallel to the adsorbing surface. While for a two-dimensional semiflexible polymer the doubling of the decay length ($2\ell_{p}$) of orientational correlations can be understood simply from the fact that in d=2 there is a single direction orthogonal to the tangent vector of a chain, in d=3, there are two orthogonal directions. Since, as emphasized above, adsorbed chains are not strictly two-dimensional, it is questionable what their decay length $\ell_{p}^{eff}$ of orientational correlations actually is. Therefore, even for *L* of the same order as $\ell_{p}$, their lateral chain dimensions cannot be straightforwardly predicted from the WLC model [26,27]. For $L\gg \ell_{p}$, an additional problem arises that excluded volume matters since in d=2 chain intersection is strictly forbidden, and the crossover to d=2 SAW behavior (mean square end-to-end distance $\langle R^{2}\rangle \propto \ell_{p}^{1/2}L^{3/2}$) [28,29,30] sets in when *L* exceeds $\ell_{p}$. These problems are potentially relevant for experiments such as atomic force microscopy (AFM) studies of DNA fragments adsorbed on various substrates; see, e.g., [31,32,33,34,35,36,37].

The existing theory [15,16] refers mostly to the limit L→∞, and, being based on the WLC model, ignores excluded volume completely. In the present work, we wish to elucidate further both the effects of excluded volume and of finite chain length on the properties of adsorbed semiflexible chains, carrying out Molecular Dynamics (MD) simulations of a coarse-grained model. We have used that model in previous work [26,27] to estimate how the adsorption transition of semiflexible polymers depends on their stiffness. While our previous work focused on estimating the critical strength $\epsilon_{wall}^{cr}$ of the potential of the adsorbing wall where adsorption occurs, and the crossover of the decay length $\ell_{p}^{eff}$ from $\ell_{p}$ to $2\ell_{p}$ upon chain adsorption, we now focus on the properties of the adsorbed polymers for wall potentials chosen such that $\epsilon_{wall}>\epsilon_{wall}^{cr}$ (note that $\tau \equiv \epsilon_{wall}/\epsilon_{wall}^{cr}-1$). We expect that understanding the properties of such more or less strongly adsorbed semiflexible polymers should be useful for the interpretation of pertinent experiments.

**Figure 1:**
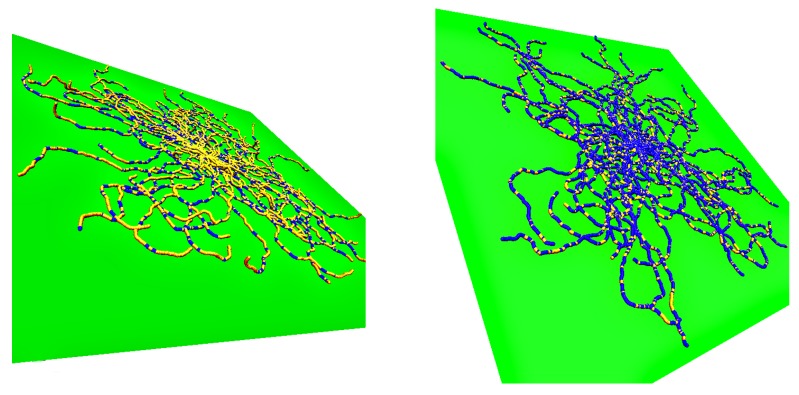
Snapshot pictures of N=50 single semiflexible polymers, described by a bead-spring model, with N=250 monomers each for the case of a chain stiffness κ=25 and wall potential depth $\epsilon_{wall}=0.60$ (**left**) and 0.80 (**right**)—see Section 2 for a precise description of the chosen model. The adsorbing surface is shown in green, and monomeric units within the range of the adsorption potential are shown in blue while those outside of this range are shown in yellow. Note that the root mean square gyration radius in the *z*-direction $\sqrt{\langle R_{gz}^{2}\rangle }$ is only about 2.14 for $\epsilon_{wall}=0.60$, and about 0.48 for $\epsilon_{wall}=0.80$, implying that in both cases the polymer conformations are almost two-dimensional while $\sqrt{{\langle R_{g}^{2}\rangle }_{\left|\right|}}\approx 48$ in both cases. All chains are grafted at the point x=0,y=0,z=0.97 so the superposition of the snapshots should not be confused with pictures of star polymers.

In Figure 1, we illustrate the above introductory discussion by snapshots for weakly adsorbed (left) or more strongly adsorbed (right) semiflexible polymers, resulting from our simulations. Note that in each figure 50 independent conformations are superimposed such that the grafting site for all chains is identical (since a single chain configuration needs not be typical, a whole ensemble of independent chains is shown in both cases). The adsorbing planar surface is shown in green, and monomers with *z* coordinate within the range of the adsorption potential are colored in blue while monomers in loops and tails (i.e., monomers outside the range) are displayed in yellow. Note that for the case of weak adsorption most of the monomers are in flat loops, i.e., the perpendicular extension of the loops from the wall amounts to a few monomer diameters only. In the case of strong adsorption, even if the majority of the monomers reside within the potential range, the polymer conformation still contains many small loops in a rather random succession along the chain.

In Section 2, we briefly summarize the model, simulation method, and describe the properties that will be analyzed. Section 3 describes our numerical results for the properties of adsorbed chains and discusses them, comparing to pertinent theoretical predictions and related simulation work when appropriate. Section 4 summarizes our findings for the distribution functions of the lengths of loops, trains and tails. Section 5 contains our conclusions.

## 2. Simulated Model

Given the fact that systems of interest such as ds-DNA involve mesoscopic length scales (where the persistence length $\ell_{p}$ is accepted to be 50 nm, meaning that about 150 base pairs correspond to one persistence length), a simulation of a chemically realistic model (with water and added salt as a solvent) is extremely difficult. As in our previous related work [26,27], we use a coarse-grained model of bead-spring type. The diameter σ of the beads is chosen to represent the effective diameter of the real wormlike polymer, e.g., σ≈2 nm in the case of (ds)-DNA. Excluded volume between the beads is described by a truncated and shifted Lennard–Jones potential,

(1) $$U\left(r\right)=4\epsilon \left[{\left(\sigma /r\right)}^{12}-{\left(\sigma /r\right)}^{6}+1/4\right],\;r<r_{c}=2^{1/6}\sigma ,$$

while for larger distances between the beads the effective potential is zero, $U(r>r_{c})\equiv 0$. This choice represents good solvent conditions (solvent molecules are not considered explicitly). The strength ϵ of the potential is used as unit of energy, ϵ≡1 (and temperature $k_{B}T=1$ as well), while henceforth σ is chosen as unit of length, σ≡1. Chain connectivity is obtained via the finitely extensible nonlinear elastic (FENE) potential between subsequent beads along the chain,

(2) $$U^{\mathrm{FENE}}\left(r\right)=-0.5kr_{0}^{2}\ln\left[1-{\left(r/r_{0}\right)}^{2}\right],\;r<r_{0},$$

with $U^{FENE}\left(r>r_{0}\right)=\infty .$ Choosing the parameters as [38] $r_{0}=1.5\sigma$, $k=30\epsilon /\sigma^{2}$ has the effect that the bond length $\ell_{b}\approx 0.97\sigma$, i.e., this does not introduce a new length scale in the problem. Other models [39] yield $\ell_{b}$ much smaller than σ and such strongly overlapping spheres may be a bit closer to reality for some semiflexible polymers but simulating stiff polymers with $L\gg \ell_{p}$ using such a model would be very difficult.

Stiffness is introduced by means of a bending potential,

(3) $$U_{b}\left(\theta_{ijk}\right)=\kappa \left[1-\cos\left(\theta_{ijk}\right)\right],$$

where $\theta_{ijk}$ is the angle that the bond vector ${\vec{u}}_{j}={\vec{r}}_{k}-{\vec{r}}_{j}$ forms with the preceding bond vector ${\vec{u}}_{i}={\vec{r}}_{j}-{\vec{r}}_{i}$, (${\vec{r}}_{i},{\vec{r}}_{j},{\vec{r}}_{k}$ being the positions of the subsequent beads). This model is a discrete counterpart of the KP model of WLCs where the coordinate *s* along the contour is a continuous variable while here only discrete positions $s_{n}=n\ell_{b}\;\left(n=1,2,\ldots \right)$ are possible. Note that for large κ one has $U_{b}\left(\kappa \right)\approx 1/2\kappa \theta_{ijk}^{2}$ so locally only small $\theta_{ijk}$ occur. The present model differs substantially from the KP model by inclusion of excluded volume (EV), but a great advantage of MD is that EV interactions (apart from nearest neighbors along the chain) can be kept or switched off to obtain a direct test for their effect. In this model, the contour length of a chain containing *N* beads is simply $L=\left(N-1\right)\ell_{b}$ while the effective persistence length, $\ell_{p}^{eff}$, characterizing the initial decay of the bond orientational correlation function, is conveniently estimated from

(4) $$\ell_{p}^{\mathrm{eff}}/\ell_{b}=-1/\ln\langle \cos\theta_{ijk}\rangle .$$

Equations (3) and (4) yield directly [26,27] for large $\kappa /k_{B}T$ when $\ell_{p}^{eff}/\ell_{b}\approx 2/\langle \theta_{ijk}^{2}\rangle$

(5) $$\ell_{p}^{eff}/\ell_{b}=\kappa /k_{B}T\;\left(d=3\right),\;\ell_{p}^{eff}/\ell_{b}=2\kappa /k_{B}T\;\left(d=2\right).$$

Equation (5) is compatible with the KP model, irrespective of whether EV is included or not.

**Figure 2:**
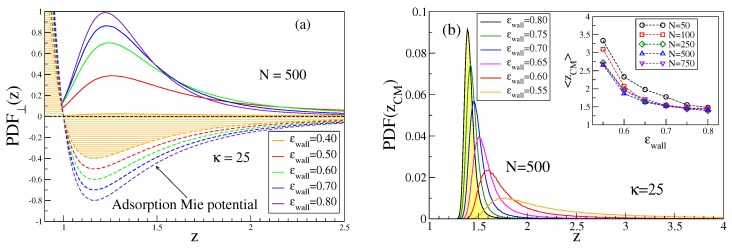
(**a**) adsorption Mie-potential, Equation (6)—broken curves, plotted for *z* and five choices of $\epsilon_{wall}$, as indicated. The corresponding monomer density distributions ρ(z) for N=500,κ=25, normalized as $\int_{0}^{\infty }\rho \left(z\right)dz=1$, are indicated by full curves, (**b**) probability distribution $P(z_{CM})$ of the center-of-mass position $z_{CM}$ of a chain with N=500,κ=25, for six choices of $\epsilon_{wall}$. Weakly bound chains ($\epsilon_{wall}=0.55$ and 0.60) have very asymmetric distributions while strongly bound chains ($\epsilon_{wall}=0.75$ and 0.80) have almost Gaussian distributions with only small asymmetry in the tails, whereas the peak position exceeds $z_{min}$ distinctly. Inset shows the variation of $\langle z_{CM}\rangle$ with $\epsilon_{wall}$ for five choices of *N*.

The adsorbing substrate is approximated by a structureless rigid wall at the plane z=0 at which the potential acts

(6) $$U_{\mathrm{wall}}\left(z\right)=\epsilon_{\mathrm{wall}}C\left[{\left(\frac{\sigma_{w}}{z}\right)}^{10}-{\left(\frac{\sigma_{w}}{z}\right)}^{4}\right],\;$$

where the constant $C=\frac{5}{3}{\left(\frac{5}{2}\right)}^{2/3}$ so that the minimum at $z=z_{min}={\left(\frac{5}{2}\right)}^{1/6}$ has the depth $-\epsilon_{wall}$, cf. Figure 2a. The potential in Equation (6), the so-called “Mie potential”, can be thought of resulting from integrating a 12–6 Lennard-Jones potential over all the atoms of an (infinite) two-dimensional plane. The resulting attraction, decaying proportional to $z^{-4}$, hence, is realistic when polymers are adsorbed on quasi-twodimensional membranes, graphene sheets, etc., or when a surface of a three-dimensional substrate is coated with a suitable surfactant monolayer to control the wettability conditions [40].

We show in Figure 2a the corresponding distributions of the monomer density for a typical case, for which the adsorption transition is predicted to occur at [26,27] $\epsilon_{wall}^{cr}=0.47\pm 0.01$. Choosing $\epsilon_{wall}$ as our control parameter, this means that Figure 2a includes both ‘mushrooms’, just before the onset of adsorption ($\epsilon_{wall}=0.40$), chains in the transition regime ($\epsilon_{wall}=0.5÷0.6$), and strongly adsorbed chains ($\epsilon_{wall}=0.7÷0.8$). As far as in all simulations the first monomer of a chain is “anchored” at the surface, we choose $z_{1}=0.97$. In addition, for the mushrooms, there occurs an enhanced monomer density near the surface while a tail of ρ(z) extends to large *z*. For strongly adsorbed chains, however, almost all monomers reside in the range 1≤z≤2.4. Monomers are seldom found in the region 0<z<1 where the wall potential is repulsive.

Apparently, the position of the minimum of $U_{wall}\left(z\right)$ does not coincide with the position of the maximum of the monomer density distribution. Actually, most of the monomers are localized further away from the wall, even if the whole chain is strongly bound to the wall. This fact becomes evident when we examine the distribution of the center of mass position of the chain, Figure 2b. It is, therefore, of interest how the center of mass position, $\langle z_{CM}\rangle$, and its distribution function, $P(z_{CM})$, depend on the parameters $\epsilon_{wall},N$, and κ, Figure 2b. Evidently, $\langle z_{CM}\rangle$ decreases with increasing $\epsilon_{wall}$ only rather slowly and shorter chains such as N=50,100 are rather loosely bound to the attractive surface, compared to the longer ones. While Figure 2b refers to κ=25 as far as in the framework of our coarse-grained model this case might mimic ds-DNA, it is clear that for the rigid rod limit and large *N*$\langle z_{CM}\rangle$ would only be slightly enhanced beyond $z_{min}$ (see Appendix A). Such long rods would undergo almost harmonic fluctuations of their position around $z_{min}$ as well as accompanying very small fluctuations of their orientation. Apparently, semiflexible polymers behave very differently from hard rods in this respect: the latter have a $P(z_{CM})$, which resembles a Gaussian, centered at $z=z_{min}$ with a width scaling as $\langle z_{CM}^{2}\rangle -{\langle z_{CM}\rangle }^{2}\propto 1/L$.

It is convenient to define a range Δz of the adsorption potential from the condition that $U_{wall}\left(z\right)<-\epsilon_{wall}/2$, which yields Δz≈0.55. This parameter allows for making comparisons with analytical work [15,16], where as adsorption potential a square well potential of depth *u* and range Δ is used. Since our choice of Δ is somewhat arbitrary, we define an adsorbed monomer fraction as

(7) $$f=\int_{0}^{\infty }dz\;U_{\mathrm{wall}}\left(z\right)\;\rho \left(z\right)/\int_{0}^{\infty }dz\;U_{\mathrm{wall}}\left(z\right),$$

rather than defining *f* from the monomers within the range of $U_{wall}\left(z\right)$ [26,27]. A similar model as Equations (1)–(6) has been used in early work [41] but only rather short chains were accessible.

While *f* can be taken as order parameter of the adsorption transition, another interesting characteristic is the orientation of the bond vectors ${\vec{u}}_{i}$ relative to the surface normal,

(8) $$\eta =\frac{3}{2}\langle \cos^{2}\zeta \rangle -\frac{1}{2}\;,$$

with ζ being the the angle between a bond vector and the *z*-axis (the average 〈…〉 includes average over all N−1 bond vectors of the chain). In addition to f,η, the mean square gyration radius $\langle R_{g}^{2}\rangle$ of the polymers is also analyzed, distinguishing perpendicular, ${\langle R_{g}^{2}\rangle }_{⊥}=\langle R_{gz}^{2}\rangle$, and parallel ${\langle R_{g}^{2}\rangle }_{\left|\right|}=\langle R_{gx}^{2}\rangle +\langle R_{gy}^{2}\rangle$ components. We note that such long wave length properties of the chains relax much more slowly than the local properties f,η, and hence it is much more difficult to both equilibrate them and obtain meaningful statistical accuracy via MD simulations [26]. We have performed multiple runs for every parameter combination, $N,\;\kappa ,\;\epsilon_{wall}$, using the HOOMD-Blue software [42,43] on graphics processing units (GPUs). For each run, N=50 chains are studied in parallel, choosing a MD time step $\delta t=0.002\tau_{MD}$ with $\tau_{MD}=\sqrt{m\sigma^{2}/\epsilon }$ (whereby the monomer mass m=1). The length of each run was at least $5\times 10^{6}\;\tau_{MD}$ and typically 6÷12 runs were carried out for N=500,750. As discussed in more detail in [26], the rapid increase of relaxation times with chain length limits the accessible range to N≤750.

## 3. Numerical Results for the Properties of Adsorbed Chains

**Figure 3:**
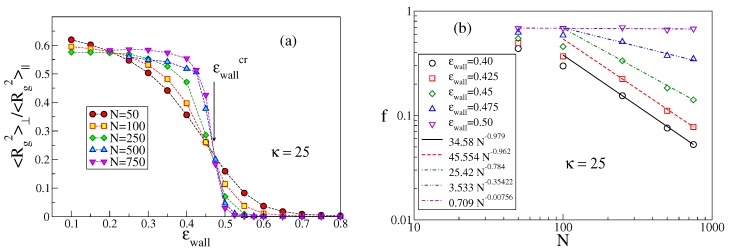
(**a**) Ratio of perpendicular and parallel parts ${\langle R_{g}^{2}\rangle }_{⊥}/{\langle R_{g}^{2}\rangle }_{\left|\right|}$ of the mean square gyration radius of semiflexible macromolecules with κ=25, plotted vs. strength $\epsilon_{wall}$ of the adsorption potential for five choices of *N*. A second order adsorption transition should show up as universal crossing point of the curves for large *N*. The resulting estimate $\epsilon_{wall}^{cr}=0.47\pm 0.01$ is indicated by an arrow, (**b**) log-log plot of the adsorbed fraction of monomers *f* vs. chain length for κ=25 and five choices of $\epsilon_{wall}$, as indicated. Broken straight lines denote power laws, the slopes show the resulting effective exponents.

The methods used to identify the critical value $\epsilon_{wall}^{cr}$ as a function of κ have already been discussed in [26,27]. For the sake of completeness, in Figure 3 we add here an example not shown previously. When the polymer chain is in the non-adsorbed mushroom state, all components $\langle R_{g\gamma }^{2}\rangle$ with γ=x,y,z are of the same order whereas in the adsorbed state $\langle R_{gz}^{2}\rangle$ should converge to a finite value as N→∞ while ${\langle R_{g}^{2}\rangle }_{\left|\right|}\propto \ell_{p}^{1/2}L^{3/2}\to \infty$, recalling that in d=2 excluded volume also matters for semiflexible chains [28,29,30] and the Flory exponents is ν=3/4 [1].

Thus, the ratio ${\langle R_{g}^{2}\rangle }_{⊥}/{\langle R_{g}^{2}\rangle }_{\left|\right|}$ for large *N* converges to a constant (which would be 1/2, if all $\langle R_{g\gamma }^{2}\rangle$ were equal) for $\epsilon_{wall}$ being less than the critical value $\epsilon_{wall}^{cr}$, whereas this ratio converges to zero for $\epsilon_{wall}>\epsilon_{wall}^{cr}$. Scaling theory [22,25] predicts that for large enough *N* right at $\epsilon_{wall}^{cr}$ the ratios ${\langle R_{g}^{2}\rangle }_{⊥}/{\langle R_{g}^{2}\rangle }_{\left|\right|}$ cross at a universal crossing point which has been estimated (for SAWs [24]) to be around 0.32. For our model and the available chain lengths, the crossing occurs, Figure 3a, near $\epsilon_{wall}^{cr}=0.47\pm 0.01$ for N≥250, while for smaller *N* the crossings are shifted to smaller values. In addition, the ordinate of the crossing occurs near 0.20±0.05 rather than near the theoretical value. We interpret these problems as being due to strong corrections to scaling. In fact, near $\epsilon_{wall}^{cr}$, the chain conformations are still of mostly three-dimensional character, and, therefore, given this choice of stiffness, excluded volume matters for very long chains only [12,13] as discussed in the Introduction.

An alternative method to search for $\epsilon_{wall}^{cr}$ is the study of the variation of *f* with *N* [25]. In the mushroom regime, only few monomers are close to the surface, and hence f∝1/N while in the regime of strong adsorption *f* should converge to nonzero values close to unity. Right at $\epsilon_{wall}=\epsilon_{wall}^{cr}$, a power law decay, $f\propto N^{1-\varphi }$, is predicted [22,23,24,25], with φ=1/2 for Gaussian chains while φ=0.48 in the presence of excluded volume [24]. In the example shown in Figure 3b, one sees that for chains that are clearly non-adsorbed (such as for $\epsilon_{wall}=0.40$), f∝1/N is verified, but only for large *N*. For chains that are clearly adsorbed (such as for $\epsilon_{wall}=0.50$), one observes undoubtedly a convergence to a nonzero value for N→∞. For intermediate values of $\epsilon_{wall}$, there is a curvature in the log-log plot, and with decreasing $\epsilon_{wall}$ the sign of the curvature changes, suggesting that for $\epsilon_{wall}=0.475$ a nonzero extrapolation is reached, while for $\epsilon_{wall}=0.45$ ultimately f∝1/N should result (although N>750 would be needed to see this).

**Figure 4:**
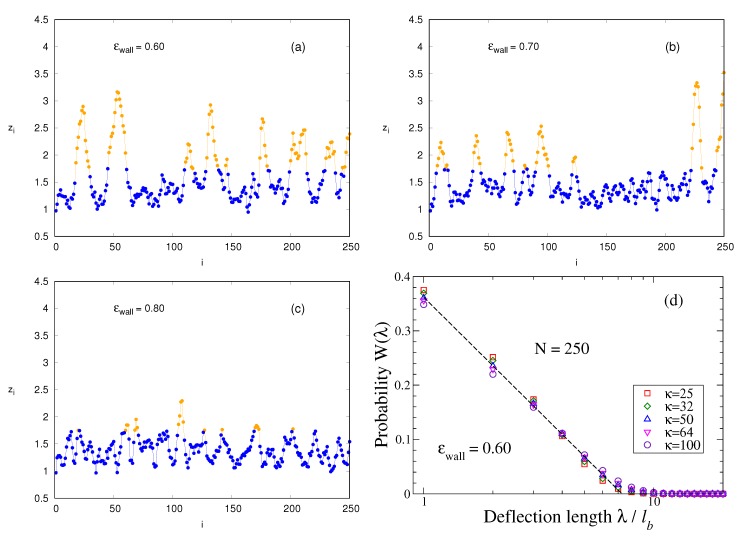
Conformations of strongly adsorbed chains (N=250,κ=25) showing the *z* coordinate $z_{i}$ of the monomers plotted vs. their index *i* labeling them along the chain contour. Three choices of $\epsilon_{wall}$ are shown: $\epsilon_{wall}=0.60$ (**a**); 0.70 (**b**); and 0.80 (**c**). Monomers in trains are shown in blue, those in loops in yellow. (**d**) probability distribution W(λ) plotted vs. deflection length λ for $N=250,\epsilon_{wall}=0.60$, and five choices of κ, as indicated. The dashed line denotes the function $W\left(\lambda \right)=0.18\ln\left(\frac{\lambda_{0}}{\lambda }\right)$ with $\lambda_{0}\approx 7.39$.

Much larger values of *N* would be required to estimate $\epsilon_{wall}^{cr}$ more precisely, too difficult to access with MD, while Monte Carlo studies where Equation (3) is used for SAWs on the simple cubic lattice allow the choice of *N* up to 25,000 [44]. However, the fact that only a discrete bond angle $\theta_{ijk}=90^{0}$ is possible on this lattice eliminates all bending fluctuations under small angles that are clearly important for polymers such as DNA, and are captured by the present model. For the lattice model, trains would simply be straight lines (typically of length $2\ell_{p}$) in the lattice plane adjacent to the surface and hence have no entropic contributions due to bending. In contrast, the present model exhibits bending fluctuations on small scales also in *z*-direction perpendicular to surface, Figure 4a–c. We see that $z_{i}$ varies monotonically from some minimum position (typically in the range $1\leq z_{i}\leq 1.3$) to a maximum $\left(z_{j}\right)$. If this maximum is still within the train, we take the distance $\lambda_{i}=\ell_{b}\left(j-i\right)$ as an entry for the distribution of the so-called *deflection* length λ [45]. This concept was introduced for a semiflexible polymer confined in a cylindrical pore of diameter $\Delta \gg \ell_{b}$. On average, the bond vectors are oriented parallel to the pore axis. However, an individual bond vector from ${\vec{r}}_{i}$ to ${\vec{r}}_{i+1}$ can make an angle $\theta_{i}$ with this axis, even though subsequent angles are strongly correlated due to stiffness. By estimating the mean square displacement $\langle {\left(z_{j}-z_{i}\right)}^{2}\rangle$ from the pore axis, resulting from adding up such misalignment (using the KP model), one finds the length scale λ, where $\sqrt{\langle {\left(z_{j}-z_{i}\right)}^{2}\rangle }=\Delta$, as $\lambda \propto {\left(\Delta^{2}\ell_{p}\right)}^{1/3}$ [45].

Qualitatively, one can take over this concept to confinement of semiflexible polymers in the potential well due to adsorption potential. There is a simple scaling argument to predict the variation of $\epsilon_{wall}^{cr}$ with $\ell_{p}$ [15]: A string of $n_{\lambda }=\lambda /\ell_{b}$ monomers, when adsorbed, wins an energy of order $\epsilon_{wall}n_{\lambda }$, whereas the entropy change due to adsorption of such a string is of order $k_{B}T$. Hence, the adsorption transition occurs when $\epsilon_{wall}^{cr}n_{\lambda }\approx k_{B}T$, i.e., $\epsilon_{wall}^{cr}\propto \ell_{b}/\lambda \propto \ell_{b}/{\left(\Delta^{2}\ell_{p}\right)}^{1/3}$ [15]. This variation has been confirmed in our previous work [26,27]. It is important to note that the standard picture of an adsorbed chain as a sequence of ”trains“, ”loops“, and (one or two) ”tails’ [21] must not be misunderstood as implying that “trains” are strictly two-dimensional chains restricted to the plane $z=z_{min}$. Such a picture does occur in the simplistic lattice model [25,44], however. There a train is a self-avoiding walk formed from straight lines (of lengths typically of order $\ell_{p}$) in the lattice plane next to the adsorbing wall, as stated above, and hence one finds $\epsilon_{wall}^{cr}\propto \ell_{b}/\ell_{p}$ for such a model [44,46,47].

It must be realized that there is only a vague analogy between the confinement of a polymer between two equivalent hard walls a distance Δ apart and the confinement caused by the attractive potential associated with a single wall within the soft “potential well” of width Δ. While in the slit pore case the average position of the center of mass of the polymer is trivially equidistant to both walls, and the monomer density distribution is symmetric around this position, the situation here is much more complicated. As we shall see, the center of mass position depends in a nontrivial way on all parameters of the problem ($N,\epsilon_{wall},\kappa$), and the monomer distribution has no symmetry properties. In addition, the confinement is by no means perfect: the formation of loops means the chain can “leak out” from the region where it is localized on average. Therefore, it is not apparent to what extent the deflection length concept can be applied here.

**Figure 5:**
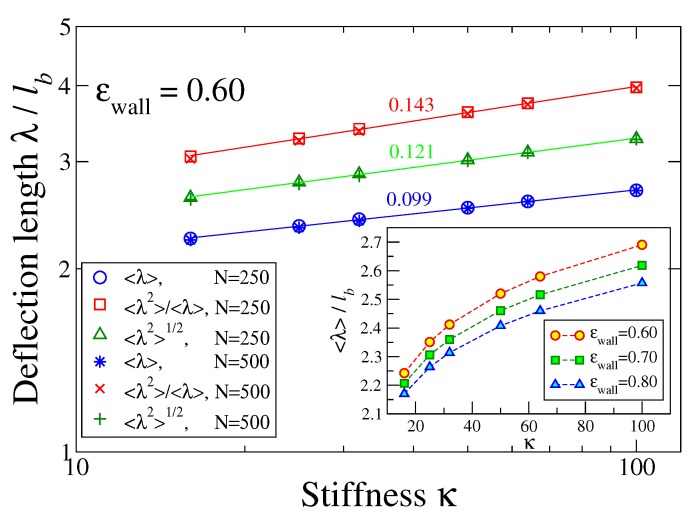
Variation of the deflection length λ with chain stiffness κ at $\epsilon_{wall}=0.60$ for chains of length N=250 and 500 in log-log coordinates, testing different definitions of the deflection length, as indicated. Slopes are displayed above the respective straight lines. The inset shows $\lambda /\ell_{b}$ vs. stiffness κ for three different strengths of $\epsilon_{wall}$ in normal coordinates.

Indeed, when we analyze configurations as shown in Figure 4a–c along the lines described above to sample the distribution function W(λ) of deflection lengths, we find a (perfectly fitted by a logarithmic function) monotonous decay of W(λ) with λ, cf. Figure 4d, whereby with increasing κ large values of λ are only slightly favored. The parameters, $A,\lambda_{0}$ in the empirical relation $W\left(\lambda \right)=A\ln\left(\lambda_{0}/\lambda \right)$, Figure 4d, depend on κ only weakly. This distribution implies that all lengths $\lambda /\ell_{b}=1,\;2,\;\ldots$, up to $\lambda_{0}/\ell_{b}\approx 7.39$, are very frequent while large values of λ are systematically suppressed. The large weight of small $\lambda /\ell_{b}=1,\;2$, etc. is clearly due to immediate reversals of the contours shown in Figure 4a–c and other small-scale structures. One could filter out such features, but such a procedure is somewhat arbitrary. Therefore, we have decided to estimate an effective deflection length from the moments of W(λ). The result is shown in Figure 5 for N=250 where the inset displays the variation of λ for three values of $\epsilon_{wall}$ as function of stiffness κ. However, as expected from the logarithmic distribution, Figure 4d, the numbers extracted from the different moments somewhat disagree with each other, and the theoretical variation proportional to $\ell_{p}^{1/3}$ is not confirmed. This problem clearly shows the limitations of the confinement analogy.

One consequence of the fact that adsorbed chains have a nontrivial structure also in the *z*-direction, Figure 4, was already studied in [26,27]. The orientational correlation function

(9) $$\langle \cos\theta \left(s\right)\rangle =\langle {\vec{u}}_{i}.{\vec{u}}_{i+s}\rangle /\ell_{b}^{2}$$

exhibits a very nontrivial variation with *s*: for s=1,2…, the initial decay is still controlled by the three-dimensional persistence length $\ell_{p}/\ell_{b}=\kappa /k_{B}T$ before a crossover to a larger value $\ell_{p}^{eff}$ sets in, i.e., $\langle \cos\theta \left(s\right)\rangle =A\exp\left(-s\ell_{b}/\ell_{p}^{eff}\right)$. For $\kappa /k_{B}T\gg 1$ and $\epsilon_{wall}\gg \epsilon_{wall}^{cr}$, one has A→1 and $\ell_{p}^{eff}/\ell_{b}=2\kappa /k_{B}T$, as expected from Equation (5). If these inequalities are not fulfilled, the amplitude *A* is somewhat smaller than unity, and $\ell_{p}^{eff}/\ell_{b}$ is also reduced in comparison to its theoretical value of Equation (5). Moreover, for $s\ell_{b}\geq \ell_{p}^{eff}$, a slow crossover to the power law decay $\langle \cos\theta \left(s\right)\rangle \propto s^{-1/2}$ [28,29,30] takes place (for s≪N). The latter regime is due to excluded volume effects, and hence out of scope of the KP model treatment [15,16].

**Figure 6:**
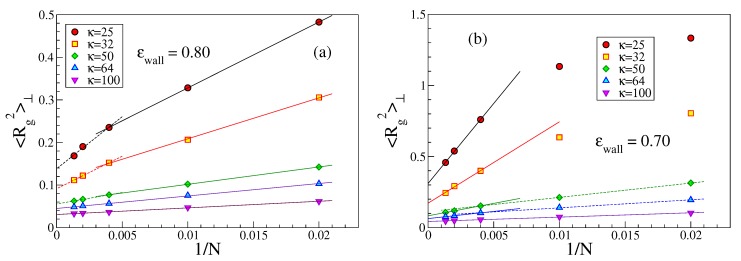
Plot of ${\langle R_{g}^{2}\rangle }_{⊥}$ vs. 1/N for five choices of κ, and $\epsilon_{wall}=0.80$ (**a**); and 0.70 (**b**). Straight lines through the data are guides for the eye.

**Figure 7:**
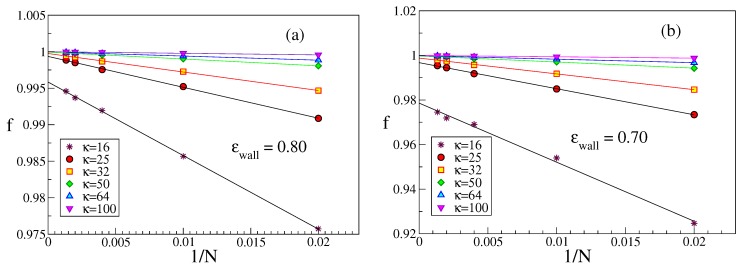
Plot of *f* vs. 1/N for $\epsilon_{wall}=0.80$ (**a**); and 0.70 (**b**). Straight lines through the data are tentative extrapolations. Six values of κ are included, as indicated. κ=16 data without excluded volume are systematically larger, while for larger κ presence or absence of excluded volume does not cause appreciable difference.

**Figure 8:**
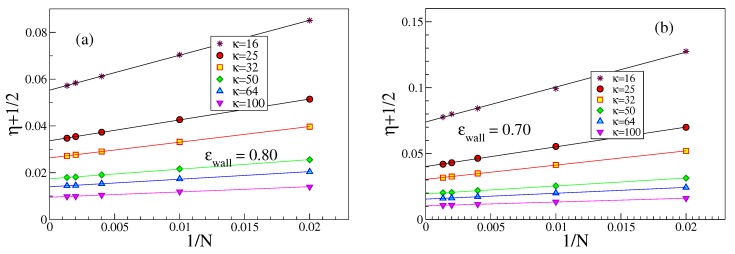
Deviation of the bond orientational order parameter η from value −1/2, i.e., from perfectly parallel orientation, plotted vs. 1/N for 6 stiffnesses κ, as indicated, for $\epsilon_{wall}=0.80$ (**a**), and $\epsilon_{wall}=0.70$ (**b**). Presence or absence of excluded volume interaction does not cause any difference on the data. Straight lines show tentative extrapolations.

An interesting issue is also the perpendicular linear dimension ${\langle R_{g}^{2}\rangle }_{⊥}$ of strongly adsorbed chains. While a rather short strongly adsorbed chain (which is like a flexible rod) cannot have any loops in contrast to much longer chains, one might expect that ${\langle R_{g}^{2}\rangle }_{⊥}$ increases with growing *N* albeit Figure 6 demonstrates the opposite trend. In addition, when N≫2κ (i.e., $L\gg \ell_{p}^{eff}$), the variation seems a bit steeper than for the case where *L* and $\ell_{p}^{eff}$ are comparable. The explanation for this unexpected behavior is that for the same choice of κ and $\epsilon_{wall}$ a longer chain is more strongly bound to the wall than a shorter one. This emerges from a study of both *f*, Figure 7, and η, Figure 8. While near $\epsilon_{wall}^{cr}$ the presence (or absence) of excluded volume does make a difference with respect to both *f* and η, and in some cases a plot vs. 1/N then shows a non-monotonic variation, this is not the case here. For stiff chains and $\epsilon_{wall}\gg \epsilon_{wall}^{cr}$, *f* is extremely close to its saturation value f=1, which means that the effect of loops is completely negligible.

**Figure 9:**
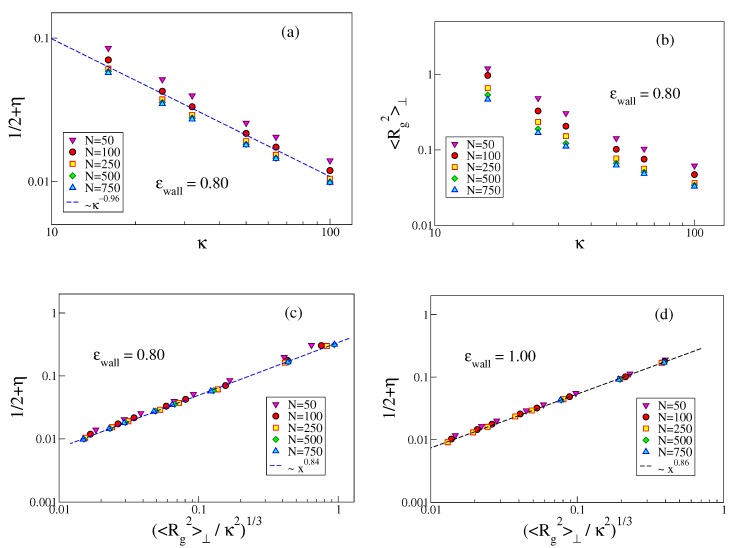
(**a**) log-log plot of $3/2\langle \alpha^{2}\rangle =1/2+\eta$ vs. stiffness κ at $\epsilon_{wall}=0.80$ for several choices of *N*, as indicated. Only data for κ≥16 are included so as to restrict attention to the strongly adsorbed case; (**b**) the same as (**a**), but for ${\langle R_{g}^{2}\rangle }_{⊥}$ vs. κ; (**c**) log-log plot of 1/2+η vs. ${\left({\langle R_{g}^{2}\rangle }_{⊥}/\kappa^{2}\right)}^{1/3}$ for $\epsilon_{wall}=0.80$; (**d**) the same as in (**c**), but for $\epsilon_{wall}=1.00$.

We note that the order parameter $\eta =(3\langle \cos^{2}\theta \rangle -1)/2$ can be written in terms of the complementary angle α=π/2−θ that a bond makes with the surface plane, as $\eta =3\left(\langle \sin^{2}\alpha \rangle -1\right)/2\approx \left(3\langle \alpha^{2}\rangle -1\right)/2$, and hence $1/2+\eta \approx 3\langle \alpha^{2}\rangle /2$, when $\langle \alpha^{2}\rangle$ is small. It is also interesting to study both $\langle \alpha^{2}\rangle$ and $\langle R_{gz}^{2}\rangle$ vs. κ at fixed *N* for the case of strong adsorption, Figure 9. It is seen that both quantities vary proportionally to $\kappa^{-1}$ within reasonable error limits. The residual dependence on *N* almost disappears when ${\left({\langle R_{g}^{2}\rangle }_{⊥}/\kappa^{2}\right)}^{1/3}$ is plotted against $\langle \alpha^{2}\rangle$, see Figure 9c.

We now turn to the interpretation of the apparent power laws visible in Figure 9. Naively, one might think that the problem is fully analogous to the problem of a semiflexible polymer confined between two hard walls a distance *H* apart, for which one finds [48] $\frac{1}{2}+\eta =\frac{1}{2}C_{\left|\right|}{\left(H/\ell_{p}\right)}^{2/3}$, with $C_{\left|\right|}\approx 0.5484$. However, using H=Δ would yield $\frac{1}{2}+\eta \propto \ell_{p}^{-2/3}$, while the data on Figure 9a rather support $\frac{1}{2}+\eta \propto \ell_{p}^{-0.96}$.

Thus, we must assume that the actual region where the monomers are localized should not be identified with a constant region H=Δz, but must be clearly of the same order as $\sqrt{{\langle R_{g}^{2}\rangle }_{⊥}}$. Thus, we would conclude

(10) $$\frac{1}{2}+\eta \propto {\left({\langle R_{g}^{2}\rangle }_{⊥}/\;\kappa^{2}\right)}^{1/3}.$$

Figure 9 shows that indeed $\frac{1}{2}+\eta$ is compatible with a power law of ${\langle {(R_{g}^{2}\rangle }_{⊥}/\kappa^{2})}^{1/3}$, and while both $\frac{1}{2}+\eta$ and ${\langle R_{g}^{2}\rangle }_{⊥}$ reveal individually a pronounced dependence on *N*, see Figure 9a,b, the plot of $\frac{1}{2}+\eta$ vs. ${\left({\langle R_{g}^{2}\rangle }_{⊥}/\kappa^{2}\right)}^{1/3}$ leads to an almost perfect collapse of the different chain lengths on a master curve. This master curve, however, is not the equation predicted above, but rather $\frac{1}{2}+\eta \propto {\left({\langle R_{g}^{2}\rangle }_{⊥}/\kappa^{2}\right)}^{0.28}$. We have no explanation of this (effective?) exponent yet. While from Figure 6b it is clear that short chains (for a given choice of κ and $\epsilon_{wall}$) are less strongly adsorbed than longer ones, the almost perfect collapse of $\frac{1}{2}+\eta$ versus ${\langle R_{g}^{2}\rangle }_{⊥}$ where the chain length dependence is eliminated (Figure 9c,d) seems nontrivial to us.

## 4. Distributions of Trains and Loops

**Figure 10:**
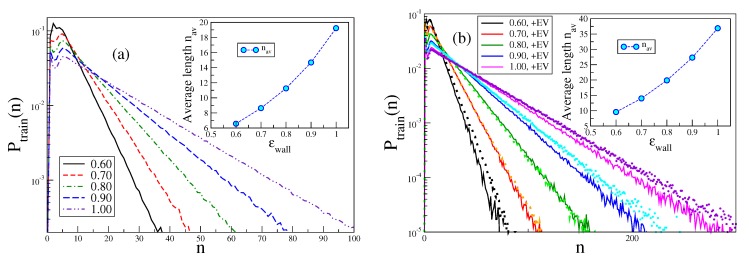
Probability distribution $P_{train}\left(n\right)$ to observe trains having a length of *n* monomers, for N=750 and two choices of κ, κ=5 (**a**); and κ=8 (**b**). Five choices of $\epsilon_{wall}$ are included, as indicated. In case (**b**), symbols indicate data with EV shut off while curves show data including EV. Inserts show the average train length, defined from $n_{av}=\int dnP_{\mathrm{train}}\left(n\right)n.$

The very interesting behavior of loops and tails in the vicinity of the adsorption threshold, where the lateral correlation length (i.e., the typical maximal lateral size of loops) is larger than $\ell_{p}$, has already been discussed in the literature [15,20]. Here, we focus rather on the behavior of trains and loops in the strongly adsorbed phase. However, since for large κ the probability to observe loops and tails is extremely small, we focus here on chains with moderate stiffness, κ=5 and 8, respectively. In these cases, the adsorption transition for N→∞ occurs near $\epsilon_{wall}^{cr}\approx 0.65$ [26,27]. Figure 10 demonstrates (note the logarithmic ordinate scale) that for large *n* the data for $P_{train}\left(n\right)$ are very well described by a simple exponential decay. The average train length increases strongly with $\epsilon_{wall}$ (but for the chosen parameters $n_{av}\ll N$, so there should not be too strong effects due to the finite length of the chain present).

**Figure 11:**
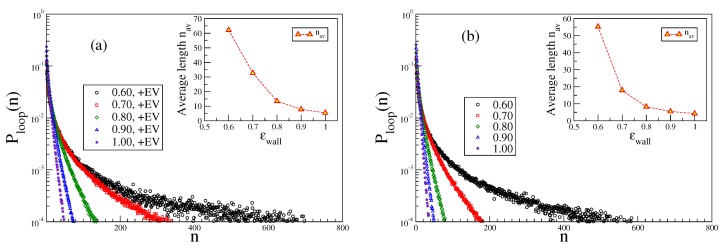
Probability distribution $P_{loop}\left(n\right)$ to observe loops having a length of *n* monomers, for N=750 and two choices of κ, κ=5 (**a**); and κ=8 (**b**). Five choices of $\epsilon_{wall}$ are included, as indicated. In case (**b**), symbols indicate data with EV shut off while curves show data including EV. Insets show the average loop length, defined as $n_{av}=\int dnP_{loop}\left(n\right)n$.

In contrast, the distribution of loop lengths $P_{loop}\left(n\right)$ exhibits a very different behavior, cf. Figure 11. There is a rapid initial decay and then a gradual crossover to a much slower decay. We tentatively interpret this as qualitative evidence for Semenov’s [15] prediction that not too far away from the adsorption transition there should be a coexistence of small loops, whose extension away from the plane z=0 is of the order of $\ell_{p}$ or less, with much fewer large loops which make much larger extensions away from the surface. Note that with excluded volume the loops are somewhat larger than without. Of course, the average length of the loops increases when $\epsilon_{wall}$ decreases towards $\epsilon_{wall}^{cr}$, reflecting the critical divergence of the correlation length of the adsorption transition in the limit N→∞. Due to the finiteness of N=750, this divergence is rounded off, however.

**Figure 12:**
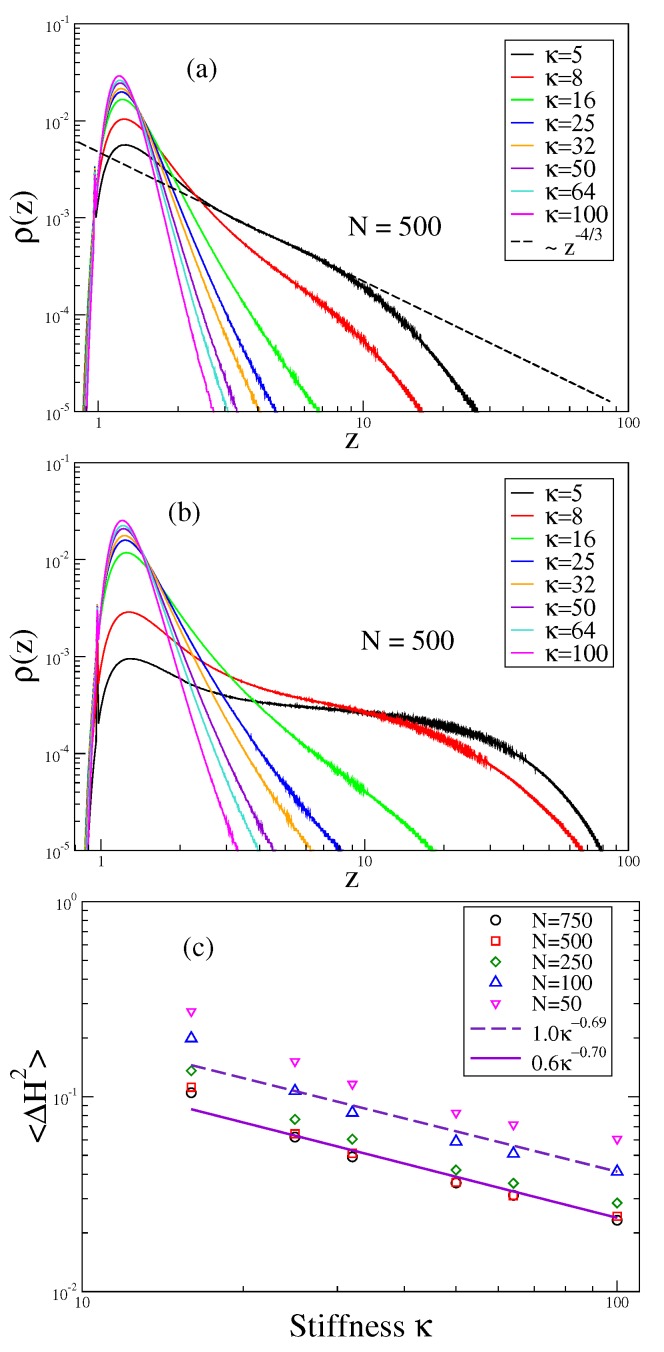
Density distribution function ρ(z) vs. *z* for several choices of κ for N=500 and $\epsilon_{wall}=0.8$ (**a**) and $\epsilon_{wall}=0.65$ (**b**); For κ=5, the predicted decay proportional to −4/3 is seen; (**c**) log-log plot of the mean square width $\langle \Delta H^{2}\rangle$ of the density distribution versus stiffness κ, at $\epsilon_{wall}=1.0$ and five choices of the chain length *N*. Tentative phenomenological power law fits are indicated by a full straight line and a broken straight line as explained in the legend.

In this context, it is of interest to study simply also the decay of the density distribution ρ(z) as a function of the distance from the surface, Figure 12. For large values of $\epsilon_{wall}$ and large κ, there is a sharp peak near $z=z_{min}$, and the mean square width of this peak, $\langle \Delta H^{2}\rangle =\int z^{2}\rho \left(z\right)dz-{\left(\int \rho \left(z\right)dz\right)}^{2}$, is extremely small and strongly decreases as κ→∞, Figure 12. Then, the polymers are rigid rods (of finite length $L=\left(N-1\right)\ell_{b}$) very tightly bound to the wall. Fluctuations of their center-of-mass position and orientation will also vanish as N→∞ (see Appendix A). However, when κ is small enough so that the chosen value of $\epsilon_{wall}$ is close to $\epsilon_{wall}^{cr}$, $\langle \Delta H^{2}\rangle$ becomes larger than unity due to a shoulder of ρ(z) for *z* much larger than $z_{min}$. Ultimately, in a medium regime, $z_{min}\ll z\ll \ell_{p}$, for $\epsilon_{wall}\approx \epsilon_{wall}^{cr}$, a power law decay $\rho \left(z\right)\propto z^{-4/3}$ is predicted to occur [15], and our data are qualitatively compatible with this prediction, Figure 12a. However, a chain length N=500 is still by far too small to yield a wide enough region $\ell_{b}<z<\ell_{p}<\sqrt{{\langle R_{g}^{2}\rangle }_{⊥}}$, which is needed to clearly observe this regime. For large *z*, ρ(z) decays exponentially with *z* for $\epsilon_{wall}>\epsilon_{wall}^{cr}$, as predicted [15]. The phenomenological power laws indicated for the variation of $\langle \Delta H^{2}\rangle$ with κ remain to be explained, however.

## 5. Conclusions

In this paper, we have studied adsorption of semiflexible polymers on a flat unstructured surface, using a coarse-grained bead-spring model, applying Molecular Dynamics methods, and varying the persistence length over a wide range. A distinguishing feature is the choice of 10–4 adsorption potential, Equation (6), qualitatively reasonable for adsorption on a thin rigid membrane. Chain lengths are used in the range from N=50 to N=750 effective monomers. Unlike flexible chains, where in the regime of weak adsorption (i.e., adsorption energy $\epsilon_{wall}$ slightly exceeding the threshold $\epsilon_{wall}^{cr}$) “pancakes” formed from blobs of mesoscopic thickness occur, we find here a thin adsorbed layer with thickness comparable to the chain thickness (or, monomer diameter, respectively). The position of the center of mass of this layer does not coincide with the position $z_{min}$ of the minimum in the adsorption potential but is clearly farther away from the adsorbing substrate surface. With increasing chain length *N* (for otherwise identical parameters $\epsilon_{wall}$ and stiffness κ), the binding of the chain to the substrate becomes tighter, i.e., the perpendicular linear dimension ${\langle R_{g}^{2}\rangle }_{⊥}$ and center-of-mass position $\langle z_{CM}\rangle$ decrease, other parameters (adsorbed fraction *f* and orientational order parameter η) come close to their saturation values, and the chain gradually adopts a quasi two-dimensional conformation. However, in no case do we come close to a situation where the polymer is strictly two-dimensional and confined to the plane $z=z_{min}$. The d=2 Kratky–Porod model never yields a perfect representation of our results for the range of stiffness κ and chain length *L* accessible in our work.

While many of our findings are in qualitative agreement with the theoretical predictions of Semenov [15], a quantitative comparison was not sought since the latter work addresses the region $L\gg \ell_{p}$, taking also $\ell_{p}$ very much larger than the effective chain thickness. This regime is not easily accessed by Molecular Dynamics simulations, however. Many predictions of the theory [15] refer to the vicinity of $\epsilon_{wall}^{cr}$, which can only be tested, if much longer chains are accessible. The theory [15] did not discuss many of the phenomena found here such as the distribution of the center of mass of the chains and its behavior with $\epsilon_{wall},\kappa$ and *N*, however, cf. Figure 2b.

Thus, the findings here suggest that additional theoretical studies addressing such issues would be desirable. With respect to the experiment, our simulations provide overwhelming evidence that properties of an adsorbed semiflexible chain are by no means identical to properties of a strictly two-dimensional chain that “lives” in the plane $z=z_{min}$, an assumption, which is implicit in many discussions in the literature. An adsorbed chain still has degrees of freedom due to its intrinsically three-dimensional character, and it is still an open problem to understand all consequences of this fact.

## Appendix A. The Hard Rod Limit

In the hard rod limit for large *N*, the macromolecules are almost perfectly aligned parallel to the wall at $z=z_{min}={\left(5/2\right)}^{1/6}$. Therefore, the wall potential can be expanded quadratically around the minimum,

(A1) $$U_{wall}\left(z\right)=\epsilon_{wall}C\left[{\left(\frac{\sigma }{z}\right)}^{10}-{\left(\frac{\sigma }{z}\right)}^{4}\right]=-\epsilon_{wall}+\epsilon_{wall}C^{\prime }{\left(z-z_{min}\right)}^{2},$$

using $C=\left(\frac{5}{3}\right){\left(\frac{5}{2}\right)}^{2/3}$ and σ=1 to find

(A2) $$C^{\prime }=8{\left(\frac{5}{2}\right)}^{2/3}\approx 14.7361$$

Displacing a rod with *N* monomers by a distance $z-z_{min}$ away from $z_{min}$ hence costs an energy

(A3) $$\Delta E=N\epsilon_{wall}C^{\prime }{\left(z-z_{min}\right)}^{2}.$$

Since such a translation is a single degree of freedom, straightforward application of the equipartition theorem $\left(\Delta E=k_{B}T/2\right)$ yields

(A4) $$\langle {\left(z-z_{min}\right)}^{2}\rangle =\frac{k_{B}T}{\epsilon_{wall}2NC^{\prime }}.$$

For N=50, this yields at $\epsilon_{wall}/k_{B}T=0.80$

(A5) $$\sqrt{\langle {\left(z-z_{min}\right)}^{2}\rangle }\approx 0.0291.$$

Thus, in Figure 2, the position of the center of mass of this rigid rod will typically fluctuate from z≈1.136 to z≈1.194. These numbers clearly show that the harmonic approximation is still justified (and it is a forteriori justified for larger *N*). Hence, in the rigid rod limit, the distribution of the center of mass is a Gaussian centered at $z_{min}$ with half width compatible with Equations (A2) and (A4).

The hard rod has a second degree of freedom where its axis is inclined by an angle α with respect to the case when all its monomers are at $z=z_{min}$. We assume for simplicity that the center of mass is still at $z=z_{min}$ and that *N* is odd so the monomer positions are ${\tilde{z}}_{i}=\pm i\;\alpha \;\ell_{b},\;i=1,\;2,\;\ldots ,\;\left(N-1\right)/2$ with $\left({\tilde{z}}_{i}=z_{i}-z_{min}\right)$ ($\ell_{b}=1$ is the distance between beads), labeling the monomer that coincides with the center of mass as i=0.

The involved energy cost is then

(A6) $$\Delta E=2\sum_{i=1}^{(N-1)/2}C^{\prime }\epsilon_{wall}\;i^{2}\;\alpha^{2},$$

which we can approximate as an integral along the contour (*L* is measured in units of σ=1)

(A7) $$\Delta E=2\epsilon_{wall}C^{\prime }\;\alpha^{2}\int_{0}^{L/2}s^{2}ds=\frac{2}{3}\epsilon_{wall}C^{\prime }\;\alpha^{2}{\left(\frac{L}{2}\right)}^{3}=\frac{1}{12}C^{\prime }\epsilon_{wall}\;\alpha^{2}L^{3}.$$

Using equipartition again, $\langle \Delta E\rangle =k_{B}T/2$, we conclude

(A8) $$\frac{1}{2}k_{B}T=\frac{L^{3}}{12}C^{\prime }\epsilon_{wall}\langle \alpha^{2}\rangle ,\;\langle \alpha^{2}\rangle =6\frac{k_{B}T}{\epsilon_{wall}C^{\prime }L^{3}}.$$

For $L=\left(N-1\right)\ell_{b}\approx 49$ and $\epsilon_{wall}/k_{B}T=0.80$, we find thus

(A9) $$\langle \alpha^{2}\rangle \approx 4.33\times 10^{-6}.$$

Hence, in the rod limit, the orientational order parameter η deviates from its saturation value η=−1/2 by at most a few parts in a million only. The order parameter f=1 is completely saturated within numerical precision.

Note also that

(A10) $$\langle R_{gz}^{2}\rangle =\frac{2}{N}\sum_{i=1}^{(N-1)/2}\langle {\tilde{z}}_{i}^{2}\rangle =\frac{2}{N}\ell_{b}^{2}\langle \alpha^{2}\rangle \sum_{i=1}^{(N-1)/2}i^{2}=\frac{1}{12}\ell_{b}^{2}\langle \alpha^{2}\rangle \left(N^{2}-1\right)$$

and hence

(A11) $$\langle R_{gz}^{2}\rangle \approx \frac{1}{2}\frac{k_{B}T}{\epsilon_{wall}}\ell_{b}^{2}\frac{1}{C^{\prime }N}\;,$$

i.e., we find that in the rod limit $\langle R_{gz}^{2}\rangle$ varies proportional to 1/N.

## References

[1] Grosberg A., Khokhlov A.R. (1994). Statistical Physics of Macromolecules.

[2] Rubinstein M., Colby R.H. (2003). Polymer Physics.

[3] Kratky O., Porod G. (1949). Diffuse small-angle scattering of x-rays in colloid systems. J. Colloid Sci..

[4] Reisner W., Pederson J.N., Austin R.H. (2012). DNA confinement in nanochannels: Physics and biological applications. Rep. Prog. Phys..

[5] Köster S., Weitz D.A., Goldman R.D., Aebi U., Herrmann H. (2015). Intermediate filament mechanics in vitro and in the cell: From coiled coils to filaments, fibers and networks. Curr. Opin. Cell Biol..

[6] Brelsford G.L., Krigbaum W.R., Ciferri A. (1983). Liquid Crystallinity in Polymers: Principles and Fundamental Properties.

[7] Donald A.M., Windle A.H., Hanna S. (2006). Liquid Crystalline Polymers.

[8] Grosberg A.Y., Khokhlov A.R. (1981). Statistical theory of polymeric lyotropic liquid crystals. Adv. Polym. Sci..

[9] Khokhlov A.R., Semenov A.N. (1982). Liquid-crystalline ordering in the solution of long persistent chains. Physica.

[10] Egorov S.A., Milchev A., Binder K. (2016). Semiflexible polymers in the bulk and confined by planar walls. Polymers.

[11] Milchev A., Egorov S.A., Binder K., Nikoubashman A. (2018). Nematic order in solutions of semiflexible polymers: Hairpins, elastic constants, and the nematic-smectic transition. J. Chem. Phys..

[12] Moon J., Nakanishi H. (1991). Onset of the excluded-volume effect for the statistics of stiff chains. Phys. Rev. A.

[13] Hsu H.-P., Paul W., Binder K. (2010). Polymer chain stiffness vs. excluded volume: A Monte Carlo study of the crossover towards the wormlike chain model. Macromolecules.

[14] Maggs A.C., Huse D.A., Leibler S. (1989). Unbinding transition of semiflexible polymers. Europhys. Lett..

[15] Semenov A.N. (2002). Adsorption of a semiflexible wormlike chain. Euro. Phys. J. E.

[16] Deng M., Jiang Y., Liang H., Chen J.Z.Y. (2010). Adsorption of a wormlike polymer near a hard wall: Crossover between two scaling regimens. J. Chem. Phys..

[17] Waters J.T., Kim H.D. (2013). Equilibrium statistics of a surface-pinned semiflexible polymer. Macromolecules.

[18] Kampmann T.A., Boltz H.-H., Kierfeld J. (2013). Controlling adsorption of semiflexible polymers on planar and curved substrates. J. Chem. Phys..

[19] Baschnagel J., Meyer H., Wittmer J., Kulić I., Mohrbach H., Ziebert F., Nam G.M., Lee N.-K., Johner A. (2016). Semiflexible chains at surfaces: Wormlike chains and beyond. Polymers.

[20] Kampmann T.A., Kierfeld J. (2017). Adsorption of finite semiflexible polymers and their loop and tail distributions. J. Chem. Phys..

[21] Fleer C.J., Cohen-Stuart M.A., Scheutjens J.M.M., Cosgrove T., Vincent B. (1993). Polymers at Interfaces.

[22] Eisenriegler E. (1993). Polymers Near Surfaces.

[23] Netz R.R., Andelman D. (2003). Neutral and charged polymers at interfaces. Phys. Rep..

[24] Klushin L.I., Polotsky A.A., Hsu H.-P., Markelov D.A., Binder K., Skvortsov A.M. (2013). Adsorption of a single polymer chain on a surface. Effects of the potential range. Phys. Rev. E.

[25] Eisenriegler E., Kremer K., Binder K. (1982). Adsorption of polymer chains at surfaces: Scaling and Monte Carlo analyses. J. Chem. Phys..

[26] Milchev A., Binder K. (2019). Linear dimensions of adsorbed semiflexible polymers: What can be learned about their persistence length?. Phys. Rev. Lett..

[27] Milchev A., Binder K. (2020). How does stiffness of polymer chains affect their adsorption transition?. J. Chem. Phys..

[28] Hsu H.-P., Paul W., Binder K. (2011). Breakdown of the Kratky–Porod wormlike chain model for semiflexible polymers in two dimensions. Europhys. Lett..

[29] Huang A., Bhattacharya A., Binder K. (2014). Conformations, transverse fluctuations and crossover dynamics of a semiflexible chain in two dimensions. J. Chem. Phys..

[30] Huang A., Hsu H.-P., Bhattacharya A., Binder K. (2015). Semiflexible macromolecules in quasi-one-dimensional confinement: Discrete versus continuous bond angles. J. Chem. Phys..

[31] Safinya C.R., Koltover I., Raedler J. (1998). DNA at membrane surfaces: Experimental overview. Curr. Opin. Colloids Interface Sci..

[32] Maier B., Rädler J.O. (1999). Conformations and self-diffusion of single DNA molecules confined to two dimensions. Phys. Rev. Lett..

[33] Maier B., Rädler J.O. (2000). DNA on fluid membranes: A model polymer in two dimensions. Macromolecules.

[34] Moukhtar J., Fontaine E., Faivre-Moskalenko C., Arneodo A. (2007). Probing persistence in DNA curvature properties with atomic force spectroscopy. Phys. Rev. Lett..

[35] Mücke N., Klenin K., Kirmse R., Bussiek M., Herrmann H., Hafner M., Langowski J. (2009). Filamentous biopolymers on surfaces: Atomic Force Microscopy images compared with Brownian Dynamics simulations of filament deposition. PLoS ONE.

[36] Rechendorff K., Witz G., Adamcik J., Dietler G. (2009). Persistence length and scaling properties of single-stranded DNA adsorbed on modified graphite. J. Chem. Phys..

[37] Moukhtar J., Faivre-Moskalenko C., Milani P., Audit C., Vaillant E., Fontaine F., Mongelard G., Lavorel P., St-Jean F., Argoul B. (2010). Effect of genomic long-range correlations on DNA persistence length: From theory to single molecule experiments. J. Phys. Chem. B.

[38] Grest G.S., Kremer K. (1986). Dynamics of entangled linear polymer melt—A molecular dynamics simulation. Phys. Rev. A.

[39] Naderi S., van der Schoot P. (2014). Effect of bending flexibility on the phase behavior and dynamics of rods. J. Chem. Phys..

[40] Xu X.M., Vereecke G., Chen C., Pourtois G., Armini S., Verellen N., Tsai W.-K., Kim D.-W., Lee E., Lin C.-Y. (2014). Capturing Wetting States Nanopatterned Silicon. ACS Nano.

[41] Kramarenko E.Y., Winkler R.G., Khalatur P.G., Khokhlov A.R., Reineker P. (1996). Molecular dynamics simulation study of adsorption of polymer chains with variable degree of rigidity. J. Chem. Phys..

[42] Anderson J.A., Lorenz C.D., Travesset A. (2008). General purpose molecular dynamics simulations fully implemented on graphics processing units. J. Comput. Phys..

[43] Glaser G., Nguyen T.D., Anderson J.A., Liu P., Spiga F., Millan J.A., Morse D.C., Glotzer S.C. (2015). Strong scaling of general-purpose molecular dynamics simulations on GPUs. Comput. Phys. Commun..

[44] Hsu H.-P., Binder K. (2013). Effect of chain stiffness on the adsorption transition of polymers. Macromolecules.

[45] Odijk T. (1983). On the statistics and dynamics of confined or entangled stiff polymers. Macromolecules.

[46] Birshtein T.M. (1979). Theory of adsorption of macromolecules. 1. Desorption—Adsorption transition point. Macromolecules.

[47] Birshtein T.M., Zhulina E.B., Skvortsov A.M. (1979). Adsorption of polymers on solid surfaces. I Effect of chain stiffness. Biopolymers.

[48] Chen J.Z.Y. (2016). Theory of wormlike polymer chains in confinement. Progr. Polym. Sci..

[49] http://www.cost.eu.

